# Mental health problems and needs of frontline healthcare workers during the COVID-19 pandemic in Spain: A qualitative analysis

**DOI:** 10.3389/fpubh.2022.956403

**Published:** 2022-07-27

**Authors:** Roberto Mediavilla, Anna Monistrol-Mula, Kerry R. McGreevy, Mireia Felez-Nobrega, Audrey Delaire, Pablo Nicaise, Santiago Palomo-Conti, Carmen Bayón, María-Fe Bravo-Ortiz, Beatriz Rodríguez-Vega, Anke Witteveen, Marit Sijbrandij, Giulia Turrini, Marianna Purgato, Cécile Vuillermoz, Maria Melchior, Papoula Petri-Romão, Jutta Stoffers-Winterling, Richard A. Bryant, David McDaid, A-La Park, José Luis Ayuso-Mateos, RESPOND Consortium, Giovanni Corrao

**Affiliations:** ^1^Department of Psychiatry, Universidad Autónoma de Madrid (UAM), Madrid, Spain; ^2^Centro de Investigación Biomédica en Red de Salud Mental (CIBERSAM), Instituto de Salud Carlos III (ISCIII), Madrid, Spain; ^3^Instituto de Investigación del Hospital Universitario La Paz (IdiPAZ), Madrid, Spain; ^4^Research and Development Unit, Parc Sanitari Sant Joan de Déu, Barcelona, Spain; ^5^Institute of Health and Society (IRSS), Université catholique de Louvain, Brussels, Belgium; ^6^Department of Psychiatry, Hospital Universitario La Paz, Madrid, Spain; ^7^Department of Clinical, Neuro and Developmental Psychology, WHO Collaborating Centre for Research and Dissemination of Psychological Interventions, Amsterdam Public Health Research Institute, Vrije Universiteit, Amsterdam, Netherlands; ^8^Department of Neurosciences, Biomedicine and Movement Sciences, WHO Collaborating Centre for Research and Training in Mental Health and Service Evaluation, University of Verona, Verona, Italy; ^9^Sorbonne Université, INSERM, Institut Pierre Louis d'Epidémiologie et de Santé Publique, IPLESP, Research Team on Social Epidemiology, Paris, France; ^10^Leibniz Institute of Resilience Research (LIR), Mainz, Germany; ^11^School of Psychology, University of New South Wales, Sydney, NSW, Australia; ^12^Care Policy and Evaluation Centre, Department of Health Policy, London School of Economics and Political Science, London, United Kingdom; ^13^Department of Psychiatry, La Princesa University Hospital, Instituto de Investigación Sanitaria Princesa (IIS-Princesa), Madrid, Spain

**Keywords:** psychological distress, mental health, occupational health, healthcare workers (HCWs), COVID-19, free list interviews, key informant interviews, qualitative study

## Abstract

**Background:**

Healthcare workers (HCWs) from COVID-19 hotspots worldwide have reported poor mental health outcomes since the pandemic's beginning. The virulence of the initial COVID-19 surge in Spain and the urgency for rapid evidence constrained early studies in their capacity to inform mental health programs accurately. Here, we used a qualitative research design to describe relevant mental health problems among frontline HCWs and explore their association with determinants and consequences and their implications for the design and implementation of mental health programs.

**Materials and methods:**

Following the Programme Design, Implementation, Monitoring, and Evaluation (DIME) protocol, we used a two-step qualitative research design to interview frontline HCWs, mental health experts, administrators, and service planners in Spain. We used Free List (FL) interviews to identify problems experienced by frontline HCWs and Key informant (KI) interviews to describe them and explore their determinants and consequences, as well as the strategies considered useful to overcome these problems. We used a thematic analysis approach to analyze the interview outputs and framed our results into a five-level social-ecological model (intrapersonal, interpersonal, organizational, community, and public health).

**Results:**

We recruited 75 FL and 22 KI interviewees, roughly balanced in age and gender. We detected 56 themes during the FL interviews and explored the following themes in the KI interviews: fear of infection, psychological distress, stress, moral distress, and interpersonal conflicts among coworkers. We found that interviewees reported perceived causes and consequences across problems at all levels (intrapersonal to public health). Although several mental health strategies were implemented (especially at an intrapersonal and interpersonal level), most mental health needs remained unmet, especially at the organizational, community, and public policy levels.

**Conclusions:**

In keeping with available quantitative evidence, our findings show that mental health problems are still relevant for frontline HCWs 1 year after the COVID-19 pandemic and that many reported causes of these problems are modifiable. Based on this, we offer specific recommendations to design and implement mental health strategies and recommend using transdiagnostic, low-intensity, scalable psychological interventions contextually adapted and tailored for HCWs.

## Introduction

Since the beginning of the COVID-19 pandemic, healthcare workers (HCWs) from pandemic hotspots around the world have reported mental health symptoms such as anxiety, depression, acute and posttraumatic stress, and insomnia (1–5). Pre-pandemic cohort studies are lacking, and we cannot know whether these problems were more prevalent after the COVID-19 outbreak (6); however, follow-up studies suggest that they might persist for at least 1 year (7–10), leading to exhaustion and resignation (11). With new variants and surges of the virus pressuring health systems worldwide (12) and concerning evidence of job quit and turnover (13–15), reducing the mental health toll of the pandemic on essential workers remains necessary.

The initial outbreak was virulent and largely unpredictable in European pandemic hotspots, such as Spain. By March 31st, 2020–2 weeks after the start of the first national lockdown–, the Spanish regions of Madrid and Catalonia had already reported 29,840 and 19,991 confirmed cases of COVID-19 infections, and excess mortality rates in the previous week had risen to 95% and 43%, respectively (16, 17). In Madrid, where 15% of all COVID-19 cases were HCWs (18), the critical care requirements were five times higher than before the pandemic as of April 1st (19). This enormous pressure on health systems brought a worldwide call for protecting HCWs, both physically and psychologically (20). Mounting evidence soon started to identify risk factors of poor mental health among HCWs, including personal factors such as being female or having a history of mental health disorders (3, 21, 22); occupational factors such as being directly involved in the clinical care of COVID-19 patients, reporting insufficient access to personal protective equipment (PPE), or being afraid of getting infected and/or infecting loved ones (21–26); or ecological factors, such as the epidemic indicators at the local level (22). In Spain, these results rapidly transferred to specific easy-to-access programs, including hotlines, ultra-brief stress management sessions at the workplace, and psychotherapy sessions tailored for HCWs (27, 28).

Even though timely and appropriate, these mental health programs had to rely on either evidence from previous epidemics or early studies constrained by the urgency of health policies following the first COVID-19 surge. In the first case, most available data came from Asian countries affected by the SARS (29) or the Middle-East Respiratory Syndrome (MERS) (30), which hindered the transportability of their results into Europe and therefore could hardly contribute to the design and implementation of mental health strategies. Moreover, although there are evident similarities between these outbreaks and the COVID-19 pandemic, significant differences in the epidemic trajectories make extrapolation of findings challenging (31, 32). Nevertheless, findings of the mental health burden among HCWs during the COVID-19 crisis align with previous epidemics studies (33). In the second case, most studies relied exclusively on survey designs that estimated the incidence of mental health problems and generated causal models, often without approaching the full range of potential mental health problems or analyzing relevant stakeholders' perspectives. Qualitative methods offer an excellent opportunity to overcome these barriers to mental health service research and delivery (34). First, they can provide a depth understanding of relevant phenomena that complement quantitative studies. In the case of a new problem with largely unknown health-related consequences, such as the COVID-19 pandemic, this has important implications for identifying relevant mental health problems, specific mental health needs, available resources (among the target population), and barriers (to the implementation of mental health programs). Second, qualitative studies stress the subjective perspectives and views of end-users and other key stakeholders, which critically enhances knowledge transfer activities, such as designing, implementing, disseminating, or scaling-up mental health programs (35). Third, qualitative studies help generate new testable hypotheses –particularly useful if prior knowledge about the phenomenon is scarce. Last, qualitative methods may be combined with quantitative methods to conduct a process evaluation of a mental health intervention program. This process can supplement the outcome evaluation of a clinical trial by expanding the knowledge into other areas, such as why a specific intervention did (or did not) work, how it was delivered, or whether there were any barriers to its implementation (36).

This study builds into the RESPOND consortium's mission to help prepare European mental health care systems for future pandemics (www.respond-project.eu). Here, we focus on two Spanish regions (Madrid and Catalonia) dramatically affected by the COVID-19 pandemic, especially during the first outbreak. We used a qualitative research design to describe relevant mental health problems among frontline HCWs and to explore their association with determinants and consequences and their implications for mental health program design and implementation. Following recommendations to bridge evidence and practice in public health (37), we interviewed community members and key stakeholders using the Programme Design, Implementation, Monitoring, and Evaluation (DIME) protocol of the John Hopkins University (38). We adopted a constructivist inquiry paradigm during our interviews (39) and a thematic analysis approach (40) during our analyses. To explore the environmental determinants of HCWs' mental health problems, we framed our results into a social-ecological model with five levels of analysis (intrapersonal, interpersonal, organizational, community, and public policy) (41), previously used among HCWs before (42) and during (43) the COVID-19 pandemic.

## Materials and methods

### Research design overview

We conducted a two-phase qualitative study in the Community of Madrid and Catalonia (Spain) following the DIME protocol (38). During Phase 1, we conducted a series of semi-structured Free List (FL) interviews where participants had to list the problems experienced by frontline HCWs since the beginning of the pandemic in Spain. During Phase 2, we conducted Key Informant (KI) interviews with participants who were considered knowledgeable of the reported problems by FL interviewees. Using semi-structured interviews, we asked KI interviewees about the nature, causes, and effects of the problems reported during FL interviews and what should be done about them. We analyzed these data using a thematic analyses approach (40) and interpreted our results using a social-ecological model (43).

### Participants

In Spain, free, universal medical care is provided by a tax-funded decentralized National Health System. The country is divided into 17 autonomous communities that organize the service at a regional level. In this study, participants were HCWs (doctors, nurses, nursing assistants, porters, psychologists, administrative staff, and unit managers) working for the Departments of Health in the autonomous communities of Madrid and Catalonia. The Community of Madrid (capital: Madrid), with a registered population of 6,745,591 as of January 2021, had 88,717 HCWs as of October 2021. Catalonia (capital: Barcelona), with a registered population of 7,716,760 as of January 2021, had 109,346 workers as of December 2020. To be eligible, participants were HCWs who were (1) 18 years old or older, (2) on duty during the first wave of the pandemic (March 2020), (3) fluent Spanish and/or Catalan speakers, and (4) able to understand the characteristics of the study and sign the informed consent form.

The RESPOND consortium elaborated the research protocol for this study. Experience from previous studies (44–46) guided the adaptation of the DIME protocol. In Spain, interviews were conducted by a postdoctoral researcher (RM) and a Ph.D. candidate (AM-M), respectively, in Madrid and Barcelona. Both researchers were familiar with the available research on mental health and HCWs. However, they were trained in interviewing techniques following a constructivist perspective, i.e., as mere facilitators interested in understanding the problems and needs *as community members and key stakeholders understand them*. The interviewers had no prior professional or personal relationship with the interviewees.

Sampling methods and recruitment strategies differed across FL and KI interviewees (see Table 1). In Phase 1 (FL interviews), we used a two-step maximum variation sampling (MVS) technique. First, we identified potential participants from three groups of interest, namely frontline HCWs (workers involved in the direct care of COVID-19 patients during the initial pandemic outbreak), mental health experts (psychiatrists, clinical psychologists, and mental health nurses), and administrators and service planners (unit coordinators, managing directors, and other decision-makers). Next, we used a matrix to ensure that we represented men and women with different expertise from the hospital and non-hospital settings. In Phase 2 (KI interviews), we asked FL participants to provide us with names of people they considered knowledgeable of their reported problems.

**Table 1:** Recruitment strategies for FL and KI interviewees.

	**FL interviews**	**KI interviews**
Sampling	Stratified (non-probabilistic)	Snowball (non-probabilistic)
Recruiters	Stakeholders	FL interviewees
Recruitment strategy	MVS: gender, age group, expertise, and type of job	Knowledgeability

*MVS, maximum variation sampling*.

The study was conducted in line with the Declaration of Helsinki and was approved by the Ethics Review Boards at Hospital La Paz in Madrid (study ID: 4498) and Parc Sanitari Sant Joan de Déu in Barcelona (study ID: PIC-277–20). Participants did not receive compensation for their participation in the study, except for the KI interviewees enrolled in Catalonia (100€).

### Procedure and data analysis

The interviewers (RM and AM-M) arranged, conducted, and analyzed the FL and the KI interviews, closely supervised by the local senior investigators (J-MH and J-LA-M). FL interviews were conducted between December 22nd, 2020, and March 24th, 2021, and KI interviews between April 1st and May 24th, 2021. In Madrid, all interviews were done in Spanish, while in Catalonia, they were done in Catalan or Spanish indistinctly.

#### Phase 1. FL interviews

We reached out to local mental health providers who were either part of or close to the RESPOND research team to recruit FL candidates. They approached potential FL candidates and asked them for verbal consent so the interviewers could contact them. Interviews were conducted as potential FL participants were referred and signed the informed consent form.

Interviews were conducted in individual format and were recorded on audio. In Madrid, they were delivered either online (*via* Zoom or Microsoft Teams) or in-person, depending on the interviewee's preferences and the COVID-19 restrictions. In Barcelona, all interviews were conducted online *via* Zoom or Microsoft Teams. Interviews took 30–45 min. Basic non-identifying information about the respondent was recorded to maintain confidentiality (age, gender, household composition, role, years of experience, and previous experience in infectious diseases emergencies), as well as interview details (interviewer, date of the interview, and interview ID). The interviewer assigned an interview ID, who kept a secured (digital) document with the identifying key. Importantly, interview questions focused on community views rather than personal disclosures–the main question was: *What are all the problems that have affected frontline health workers living in Spain since the start of the COVID-19 pandemic?* First, the interviewer asked respondents to list as many problems as they could think of and provide a short description of each problem they identified–following the DIME protocol, the primary question did not focus only on problems directly related to mental health. The respondents were then repeatedly probed to list as many responses as possible until they indicated they could think of no more. At the end of the interview, the interviewer asked the participants to think of someone knowledgeable of the problems they mentioned and whether they could be interested in taking part in Phase 2 (KI interviews). If so, we asked them to contact the person and get verbal consent so that we could contact them to sign the informed consent form.

The FL interviews analysis consolidated all data into a single list of responses for each FL question, including the number of different interviewees reporting each response. This process was done locally by the interviewers and the supervisors the day after the last interview was conducted. The procedure was as follows. The interviewers listed all the different responses from the interview forms in Spanish/Catalan, placing the interviewee ID number next to the response. If multiple interviewees reported the same problem, all the relevant ID numbers were listed next to that response. If two or more respondents referred to the same concept but used different wording, the review team selected and recorded the wording they felt was most accurate and most likely to be understood by a member of the target population (i.e., a KI interviewee).

#### Phase 2.KI interviews

Once the FL phase was concluded, the research team met with the mental health providers who initially approached potential FL interviewees at each study site. The problems reported in the FL interviews to be included in the KI interviews were identified during the meeting. The selection was made by consensus, considering that they were (a) mental health problems, (b) frequently reported, and (c) potentially modifiable through mental health intervention programs.

The procedure for collecting and coding sociodemographic information and anonymizing data was the same as for the FL interviews. The main difference between FL and KI interviews was that the latter was less structured. During the KI interviews, the researcher first introduces the topic and asks a pre-defined ‘grand tour' question (e.g., “*In the Phase 1 interviews, some of your colleagues said that frontline HCWs were afraid of getting infected –they were worried about having COVID-19, but also about infecting their loved ones, especially at home. Could you tell me a bit more about this?”)*. Following this introductory question, the interviewer broke in only to probe for more information or guide the respondent back if they diverged from the topic. Next, the following questions were asked for each problem until the respondent had nothing further to say:

- The nature of the problem (e.g., *What are the characteristics/symptoms or signs? How is fear of infection recognized / how do you recognize someone who is afraid of getting infected?*)
- Perceived causes (e.g., *What do [frontline HCWs] generally perceive as the cause(s) of being afraid of getting infected?*)
- The effects on the person with the problem and others close to them (e.g., *What effect does fear of infection have on the person themself?*)
- What do people currently do about it (e.g., *What do [frontline HCWs] do to handle this fear of infection?*)
- What should be done about it (e.g., *What should be done with the problem of having frontline workers afraid of getting infected*)

All interviews were transcribed using an automated transcription assistant for audio data (NVivo Transcription). Next, we coded all interview transcripts using the NVivo program (NVivo 11), separately in Madrid (RM and KRM) and Barcelona (AM-M and MF-N). We did a thematic analysis to identify (a) symptoms (i.e., descriptions of the problems), (b) causes, (c) effects, and (d) actions that could be done against these problems. We included all data items that could potentially contribute to any of these categories, regardless of the moment they were mentioned during the interview (e.g., interviewees often mentioned effects when they were asked about the nature of the problem). We then performed a thematic analysis using the structure of the KI interviews as the coding frame (40). Following the DIME protocol, we first listed and calculated the frequency of each problem's different symptoms, causes, effects, and actions (38). This information was then transferred to a summary sheet and independently reviewed by pairs of researchers in Madrid (RM and KM) and Barcelona (AM-M and MF-N). After collapsing similar categories (e.g., “the clinic did not provide adequate protective equipment,” “we did not have gloves,” and “we were clearly unprotected”), the perceived causes and effects were categorized following McLeroy's (41) and Hennein and Lowe's COVID-19 social-ecological model (43), which includes five levels of analysis, namely intrapersonal, interpersonal, organization, community, and public health. If a potential cause, effect, or action, could be classified under more than one category (e.g., shortages of protective equipment could be identified as a determinant at the organization and public policy levels), we always classified it into the lowest level (e.g., organizational).

## Results

We recruited 75 participants (41 in Madrid and 34 in Catalonia) during phase 1 (FL interviews) and 22 participants (10 in Madrid and 12 in Barcelona) during phase 2 (KI interviews). Their characteristics are shown in Table 2.

**Table 2:** Characteristics of the participants.

	**Free list (FL) interviews**			**Key informant (KI) interviews**		
	**Total** **(*****n*** = **75)**	**Madrid** **(*****n*** = **41)**	**Catalonia** **(*****n*** = **34)**	**Total** **(*****n*** = **22)**	**Madrid** **(*****n*** = **10)**	**Catalonia** **(*****n*** = **12)**
**Age group, *n* (%)**						
18–35	23	14	9	7	3	4
36–50	42	17	15	14	6	8
>50	20	10	10	1	1	0
**Gender**						
Female	46	21	25	14	6	8
Male	29	20	9	8	4	4
**Job**						
Frontline worker	37	17	20	13	5	8
Mental health expert	26	16	10	8	4	4
Administrators and service planners	12	8	4			
**Facility**						
Hospital	36	17	19			
Non-hospital	27	16	11			
NA^a^	12	8	4	22	10	12

^a^Not asked to administrators and services planners (n = 12) and to KI interviewees (n = 22).

### Phase 1. FL interviews

After combining items from two or more participants that were referring to the same problem (e.g., “insomnia” and “sleeping problems”), we identified 26 problems reported by FL interviewees in Madrid and 30 problems in Catalonia. The most frequently reported problems were similar across sites. They included lack of training and experience, fear of infection, uncertainty (about the future, the epidemiological and economic situation, etc.), excessive workload, psychological distress, insufficient protective equipment, and guilt (see Table 3).

**Table 3:** Frequency of problems reported by FL interviewees.

**Madrid (*****n*** **=** **41)**	
Excessive workload	21
Fear of infection/vulnerability	21
Insufficient and conflicting information, clinical protocols, and training	17
Uncertainty	17
Insufficient protective equipment	16
Institutions do not organize and coordinate work, lack of confidence in the institutions	15
Stress, anxiety	15
Exhaustion, hopelessness	14
Loneliness, sadness, neglect	11
Emotional problems (anxiety, activation level, low mood)	10
Sleeping problems, nightmares	9
Lack of recognition/understanding [+conflict between colleagues]	9
Anger, impotence, frustration	9
Work leave/Insufficient staff	8
Work adaptations (changes in job functions/workspaces)	7
Poor quality of clinical attention	5
**Catalonia (*****n*** **=** **34)**	
Lack of information, knowledge, training, and experience	22
Fear and uncertainty	20
Excessive workload and stress	20
Lack of PPE, material, and resources	18
Guilt, helplessness, ethical dilemma, emotionally challenging situations	16
Institutions do not organize and coordinate work	8
Anxiety	7
Abandonment and lack of support from high positions	6
Loneliness and social isolation	6
Sadness and hopelessness	6

Less than 5 participants reported the following problems: sleeping problems; stigma and lack of psychological support; worsening of previous issues; interpersonal and family problems; mental exhaustion; irritability; insecurity; dysregulation of dietary habits; emotional disorders; low relevance of less urgent issues; physical exhaustion; mood shifts; separate professional and personal matters; distrust; hallucinations; the discomfort of wearing the protective equipment; bureaucratic problems; confusion; physical problems; changes at a professional level; reconciliation of work and family life; continuous exposure to death and suffering; conflicts between coworkers; prioritization decisions; loss of leadership; inadequate psychological interventions; doubts regarding severity; relationship with patients' relatives; suicidal thoughts; alienation.

### Phase 2. KI interviews

First, we reviewed the list of problems reported during the FL interviews to decide whether any should be further combined. We only combined problems that expressed the same concept, creating broader categories (e.g., emotional problems and psychological distress). Next, we selected five problems at both study locations based on whether they were (a) mental health problems, (b) frequently reported, and (c) potentially modifiable through mental health intervention programs. The final set of problems included fear of infection, psychological distress, stress, moral distress, and interpersonal conflicts among coworkers. We collected four main themes per problem and classified them using a five-level social-ecological model, including intrapersonal, interpersonal, organizational, community, and public policy levels.

#### Nature of the problem, causes, and consequences

**Figure 1:**
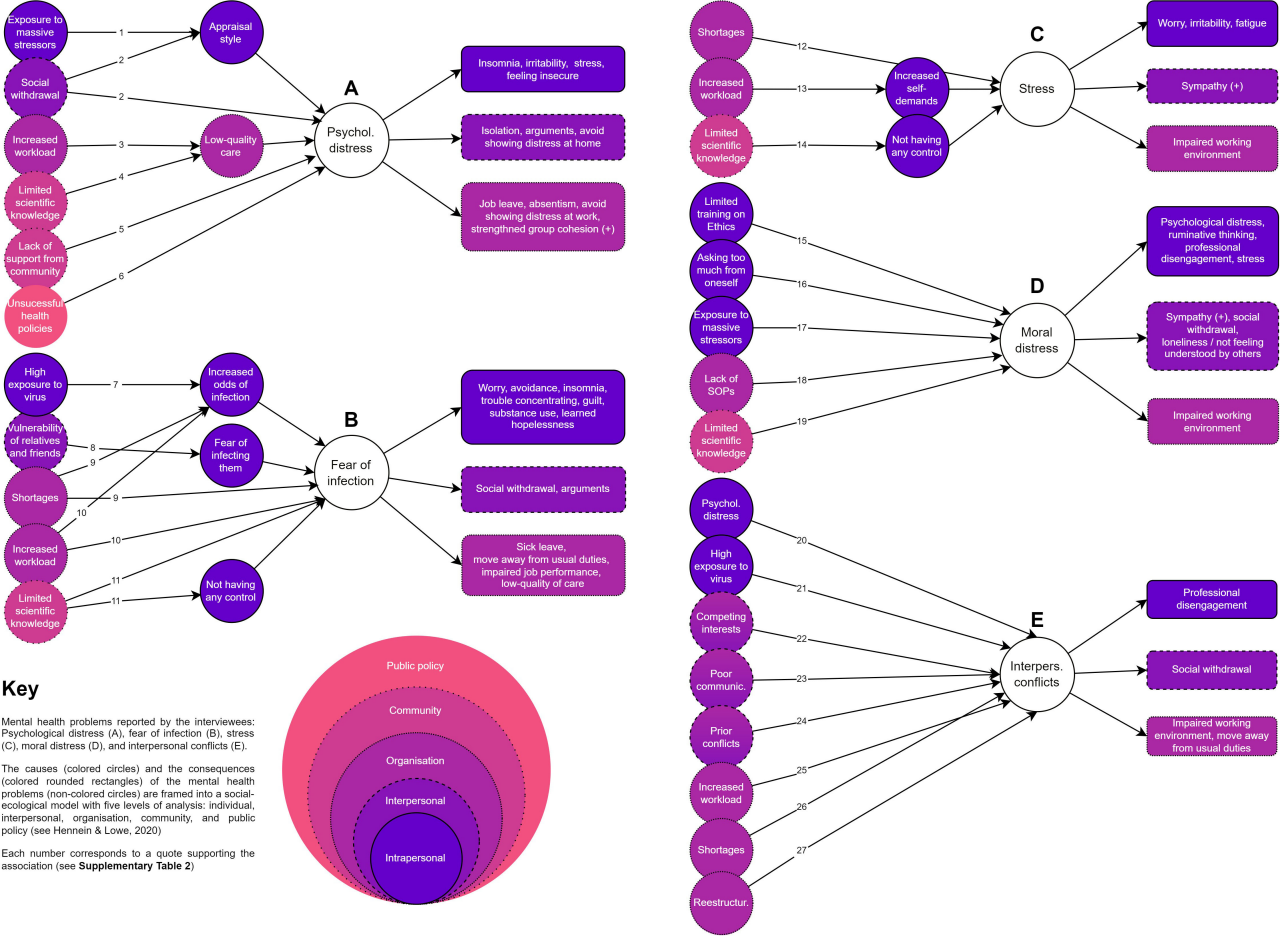
Multi-level, subjective causal models of the five mental health problems, as perceived by KI interviewees.

The first three themes included the nature of the problem (i.e., a description of the problem), their determinants (i.e., causes), and their consequences (i.e., effects) (see Figure 1 and Supplementary Table 1). Participants' identifiers used across quotes are shown in Supplementary Table 2.

##### Psychological distress

This problem (see Figure 1A) included various facets, such as arousal [“you can think about it in terms of arousal: you constantly felt in danger and witnessed how patients were being mistreated” (HULP50)], sleep problems [“In the beginning, I felt really strong […] but, when I became aware that dozens of patients were dying every day, then I started to have trouble sleeping” (HULP47)], crying [“many colleagues cried all the time because they were completely overwhelmed” (HULP47); “we cried all the time, together, but I know that other nurses asked the supervisor to let them into his office to cry alone” (HULP51)], feeling lonely [“I have seen loneliness among patients mostly, but also among HCWs; many of them lost relatives or friends that they could not even bury properly and sometimes we couldn't express our condolences in person” (HULP44)] or feeling overwhelmed by the widespread uncertainty [“I felt like a first-year medical resident because I had no idea what to do (with COVID-19 patients)” (PSSJD_10)]. One informant also emphasized something positive about being distressed: “We were all feeling very fragile and on edge, and although this was bad, it also increased the group cohesion” (HULP48).

The interviewees associated the problem of psychological distress with a wide range of factors, covering the five levels of analysis: individual, interpersonal, organization, community, and public policy. Firstly, they identified the exposure to massive stressors and social withdrawal [“In some wards, everyone died; those people had a hard time” (HULP44, quote #1); “It's been harder for workmates that have completely isolated themselves from their families” (HULP47, quote #2)]. They also mentioned that not all HCWs reacted similarly to the same stressors due to individual differences (i.e., different appraisal styles). Secondly, factors such as increased workload [“There wasn't enough time to attend adequately to all patients” (HULP47, quote #3)] and limited scientific knowledge [“What we thought we were doing well, we then found out it wasn't scientific evidence, or that further studies had proven otherwise” (PSSJD_07, quote #4)], were factors leading to low-quality care and, consequently, to psychological distress. Finally, they described a lack of support from the community [“I would do well with, for example, a working group or a team [...] where you can share your experiences and feel supported and that you are not alone” (PSSJD_01, quote #5)] and unsuccessful health policies [“The hard part of the waves [second and third] was that you continued to go to war, and you knew that the bar terrace in front of you was full of people” (HULP46, quote #6)].

Respondents related various consequences to psychological distress. At an individual level, they identified insomnia, irritability, stress, and feeling insecure. On the other hand, at an interpersonal level, they reported isolation, discussions, and distress intolerance at home. Finally, at an organizational level, they mentioned consequences such as job leave, absenteeism, and distress intolerance at work.

##### Fear of infection

Many respondents reported fear of infection (i.e., fear of contagion or fear of being the source of contamination of others; see Figure 1B). One of our KI interviewees, who served as a psychiatrist in a major hospital's emergency room during the first pandemic outbreak, said, “every HCW was afraid, which was reasonable given the circumstances” (HULP48). According to another interviewee, “you knew [when someone was worried about becoming infected] because they avoided social contact and constantly monitored symptoms and thought whether they might be infected” (HULP49). However, the feeling was very different “depending on the household composition” (HULP51) because “most people were not afraid of getting infected themselves, but of taking the virus home (…), and they were anxious and irritable” (HULP46).

Respondents related this fear to individual, interpersonal, organizational, and community factors. For instance, they identified factors such as high exposure to the virus [“I was a lot less afraid in the first wave because I wasn't with COVID patients, but now, I see people dying every day [...], and you can't help thinking that it's going to happen to you too” (HULP49, quote#7)]. Also, shortages (“We didn't have protective gear, or we didn't know how to use it” [HULP48, quote #9]) and increased workload [“There was so much work to do you couldn't even go to the bathroom” (HULP47, quote #10)]. Moreover, all of these factors increased the odds of infection for HCWs. Respondents also reported that the level of vulnerability of relatives and friends increased the fear of infecting them [“Some work colleagues lived with their elderly mother, who had diabetes and cancer, and they didn't remove their face mask, not even in bed” (HULP51, quote #8)]. Finally, having limited access to knowledge made respondents feel like they had no control [“We didn't know how to treat this kind of patients, which were so delicate” (HULP47, quote #11)].

According to the respondents, the fear of infection led to intrapersonal problems such as worry, avoidance, insomnia, trouble concentrating, guilt, substance use, and learned hopelessness; interpersonal problems such as social withdrawal and arguments with peers, friends, and family; and issues within the organization, with increased sick leaves, impaired job performance or low-quality of care.

##### Stress

Participants also reported stress (see Figure 1C). They referred to being overtaken by circumstances [“(frontline HCWs) said that they were very nervous because they were moved to other wards and had to take care of patients that they were not used to working with” (PSSJD_10)]. This situation increased their arousal levels [“HCWs were easily startled at work” (PSSJD_12)]. It also made them feel that they were not able to cope with the situation [“you forgot to do things that you would have never forgotten to do before [the pandemic] because you had a lot of things in your mind” (PSSJD_13)].

Respondents associated stress with external factors at an organizational and community level, such as shortages (“I think there are structural conditions in the hospitals, and even more so in a moment of crisis, a precariousness that leads to... I believe that stress is not only a personal problem of having resources” [PSSJD_06, quote #12]). Furthermore, from their point of view, an increased workload led to increased self-demands, which increased stress levels (“Because all of a sudden, we had an avalanche of work that we could not assimilate” [PSSJD_03, quote #13]). Finally, once again, limited access to scientific knowledge made them feel like they had no control:

What causes the problem [stress], especially in this case, is facing a novel disease and not having protocols to follow. They give you constantly changing protocols regarding syndromes and treatment; everything changes as you work. What is valid initially wasn't valid 24 o 48 h later, 2 or 3 weeks later. This led to a feeling of insecurity and stress [PSSJD_14, quote #14].

Regarding the consequences of stress, respondents identified worry, irritability, and fatigue at an individual level and an impaired working environment at an organizational level. Finally, they pointed out sympathy as a positive effect at an interpersonal level.

##### Moral distress

Moral distress (see Figure 1D), was characterized by a deep feeling of not doing enough for the patient in terms of care provision (“I felt guilty when patients called me, and I could not call them back [due to excessive workload]” [PSSJD_03]; “some colleagues were overwhelmed because patients died alone all the time, and they could do nothing” [PSSJD_10]), and closeness (“you eventually become less empathic and caring toward the patient” [PSSJD_04]. There was a “continuous questioning of standards and operating procedures” (PSSJD_08), which added to many people “taking responsibilities that exceeded their capacities (…) for instance, ICU doctors were distressed by having to decide who received mechanical ventilation and who didn't” (PSSJD_06).

Respondents related this problem to individual, interpersonal, organizational, and community factors, such as limited training on ethics:

The level of knowledge and thought regarding the practice of critical care is relatively low in ethical terms. I know this because of my experience in critical care units and hospitals. There is a low level of applied ethics in healthcare training programs, so suddenly, we found ourselves discussing whether one life had more value than another and whether relatives could come in or not. This line of questioning was seen negatively, but beyond the problem, I believe the effects were experienced as moral distress [PSSJD_06, quote #15].

Also, asking too much of yourself (“It comes from our need as health care workers to save lives; That instilled thing: I can do it, and I will save you or try to” [PSSJD_07, quote #16]); exposure to huge stressors (“The nature of this illness has been so terrible” [PSSJD_05, quote #17]); lack of Standard Operating Procedures (SOPs) (“Not having a precise protocol, often it's just that, not having a clear protocol or having it but not approving it” [PSSJD_14, quote #18]); and limited scientific knowledge (“Knowing how the disease works gives you more confidence [...] I think we don't live as much from guilt anymore, knowing that the illness has this process” [PSSJD_05, quote #19]).

According to respondents, moral distress led to personal consequences such as psychological distress, ruminating thoughts, professional disengagement, and stress. Respondents identified social withdrawal, loneliness, and not feeling understood by others at an interpersonal level. However, they mentioned sympathy as a positive effect. Finally, they identified an impaired working environment at an organizational level.

##### Interpersonal conflicts among coworkers

According to respondents:

It was a very complex problem. There were strong frictions between working groups [doctors, nurses, porters] because functions suddenly became blurry. Different levels of exposure [to the virus] generated rivalry; the higher the exposure, the greater the fear and the responsibility, but not the reward (HULP48).

This problem (see Figure 1E) was directly linked to trust (“we worked in pairs, and we had to take care of one another; if you didn't know the coworkers [because of constant redeployments and reassignments], you couldn't easily trust them” [HULP46]). This issue often results in a lack of cohesion (“when a big group of people doesn't work well together, people tend to gather in smaller groups” [HULP50]). One of its main triggers was that “some people tried to sneak out of work, and that was a strong turning point” (HULP47).

Participants associated interpersonal conflicts with several factors. At an individual level, they mentioned psychological distress (“Factors that I have mentioned to you, like fear of infection, uncertainty, or how we are feeling, affect workplace relationships” [HULP49, quote #20]) and high exposure to the virus (“In my opinion, there were levels of exposure [to the virus] that created rivalries” [HULP48, quote #21]). At an interpersonal level, they identified the following causes: competing interests (“There can be conflicting criteria (...) It's not that one is right and the other one wrong, but when two people think differently, there can be tension” [HULP49, quote #22]); poor communication (“Some times there are conflicts between workmates [...]in moments of tension where there is a lack of communication among us, conflicts and tensions arise” [HULP47, quote #23]); and prior conflicts (“[The pandemic] has uncovered tensions that used to be hidden or tolerated” [HULP48, quote #24]). At an organizational level, respondents related interpersonal conflicts to an increased workload (“[There were different levels of] involvement [...], and this created differences and tensions” [HULP48, quote #25]) and shortages (“Some workmates got mad with the supervisor, who didn't give us PPE” [HULP48, quote #26]). Also, to restructuring (“Every time we opened a new COVID one [new unit], part of the staff changed [...] there was a lack of belonging in the unit” [HULP46, quote #27]).

Ultimately, participants identified professional disengagement and social withdrawal as personal and interpersonal consequences. At an organizational level, they described an impaired working environment and the assignment of unfamiliar tasks.

#### Reported strategies to overcome the problems

**Figure 2:**
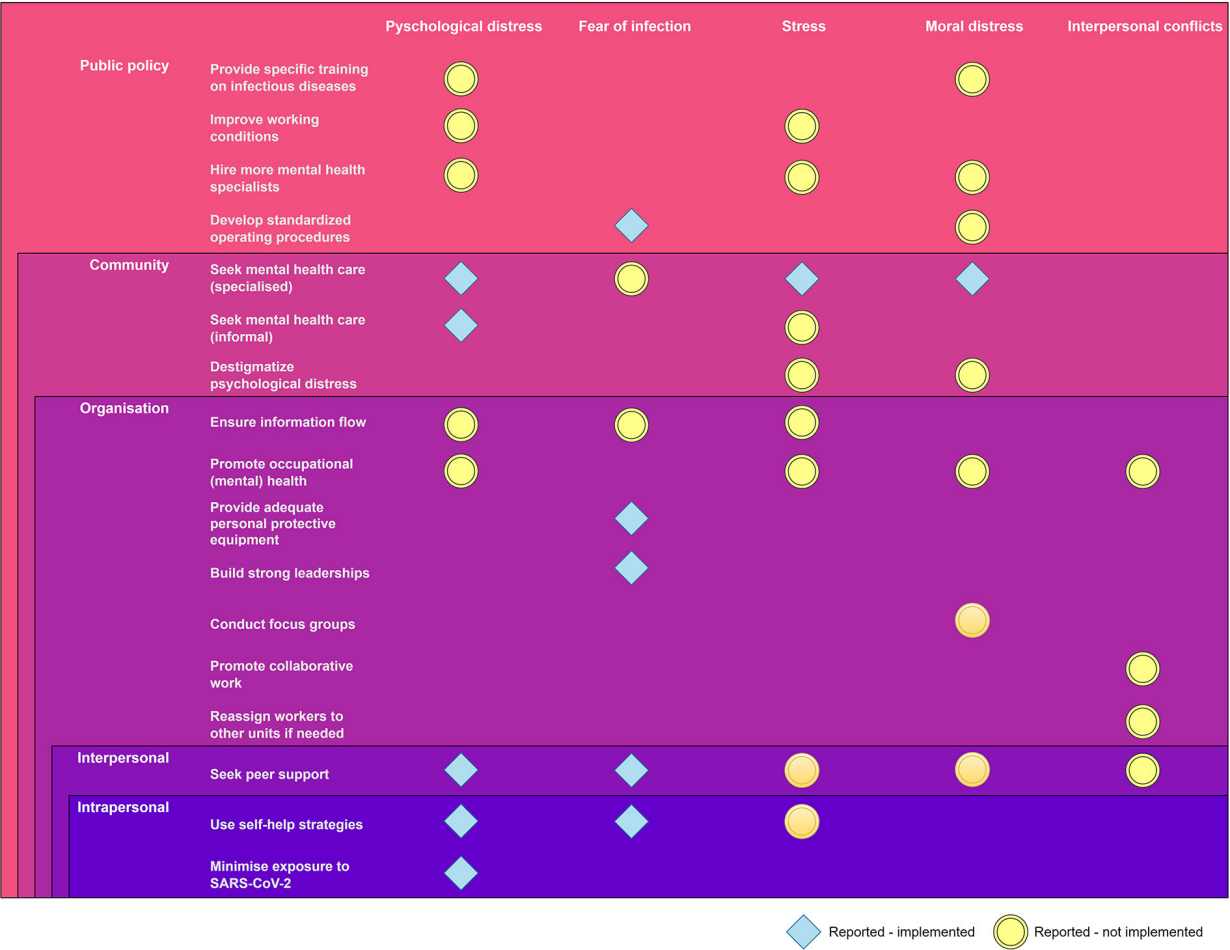
Strategies currently implemented (blue rhombus shapes) or requiring implementation (yellow circles) to improve frontline HCWs' mental health problems, as perceived by KI interviewees.

The fourth theme contained the strategies that, according to our respondents, frontline HCWs used to cope with the reported problems (see Figure 2).

At a public health policy level, respondents only identified one strategy: developing standardized operating procedures to help overcome the fear of infection. On the other hand, many strategies were already in place at the community, organizational, interpersonal, and personal levels, namely: seeking specialized mental health care to overcome psychological distress, stress, and moral distress (“They offered group mindfulness activities that helped us relax; many people enjoyed them” [HULP46]); providing adequate personal protective equipment and building strong leadership to face the fear of infection (“Someone who knew what to do would have come in handy (..) to assure us that we wouldn't be lacking PPEs” [HULP47]); and seeking peer support and using self-help strategies to reduce the impact of psychological distress (“Offer sessions on emotional containment, emotional intelligence, addressing things, and how not to take them home” [PSSJD_03]).

However, respondents highlighted that many strategies were not being implemented for specific problems (e.g., interpersonal problems), despite feeling they would be beneficial, especially at the public policy, community, and organizational levels. For instance, at a public policy level, respondents suggested strategies such as providing specific training on infectious diseases or improving HCWs' working conditions to overcome psychological distress, stress, and moral distress: “Training, training, and more training. In the case of Ebola, it helped me a lot the fact that we had a lot of training; we brought the techniques well learned” (HULP44); “There's not enough staff, and work overload is still massive (especially with this new variant, which makes patients get worse in a matter of hours” (HULP47). Furthermore, from a community perspective, they advocate destigmatizing psychological distress to face anxiety and moral distress (“Normalize the situation, you are not on your own, and many have been through the same thing as you; together, we can improve people92s resilience and resist without tearing apart” [PSSJD_08]).

At an organizational level, respondents recommend ensuring the information flow to overcome problems such as psychological distress, fear of infection, and stress:

That fear had to be dealt with by giving information on what to do to avoid getting infected. Even if we had little information about what we were facing then, they should have conveyed a sense of tranquility, for instance, saying that you were more or less protected with good hygiene (HULP47).

In addition, to face moral distress, they bet on promoting occupational mental health and organizing focus groups with peers:

To do joint therapy, we should all get together. Well, we can't right now, but get together in groups and talk about our experiences, about how we have been able to cope, how it's still affecting us, and different strategies that we have put in place to bear it as best as possible (PSSJD_07).

Regarding interpersonal conflicts, respondents felt that the organization should promote collaborative work or reassign workers to other units if they feel overwhelmed (“With supervision, we have to work more as a team, talking about the problems that arise at work. We have to consider everyone's roles, distinct responsibilities, and sensitivities” [HULP48]).

Finally, respondents identified strategies that HCWs used to deal with specific problems but considered that they should also use them with other issues. For instance, at an interpersonal level, seeking peer support to face stress, moral distress, and interpersonal conflicts, and not only to face psychological pain or fear of infection.

## Discussion

In this study, we used a qualitative research design to identify and describe relevant mental health problems among frontline HCWs and explore their association with determinants and consequences at various levels as perceived by HCWs. We interviewed stakeholders from two early pandemic hotspots (Madrid and Catalonia), including frontline HCWs, mental health providers, administrators, and service planners. Our main findings, alongside implications for future research, policy, and practice, are discussed below.

### Mental health matters—even after the first pandemic outbreak

The study aimed at understanding HCWs' problems and needs so that we could inform evidence-based mental health strategies. To that end, we first asked our FL interviewees to provide a list of problems (in general). We found that mental health problems were reported as frequently as life-threatening problems such as lack of training to avoid infection or PPE shortages, indicating that mental health problems are still considered relevant for HCWs almost 1 year after the first pandemic outbreak. This result is in line with evidence from longitudinal survey studies conducted in Spain (7, 9) and abroad (10, 47, 48), showing that poor mental health outcomes among HCWs tend to persist over time. In a country where one in two nurses had thought of quitting their job since the beginning of the pandemic (49), the increased levels of tiredness and exhaustion reported by some of our interviewees serve as a plausible explanation for this sustained poor mental health (“[we] had not been allowed to disconnect or deactivate [since the beginning of the pandemic]”; “[HCWs] have got used to sleeping 3 h a day and being tired”). A qualitative prospective study conducted in the UK, another European COVID-19 pandemic hotspot, supports this idea (11). Using consecutive in-depth interviews, the authors identified three pandemic phases, namely *emergency and mobilization* (late winter-spring 2020), *consolidation and preparation* (summer-early autumn 2020), and *exhaustion and survival* (late autumn 2020-winter 2021). This last phase is critical to our study as it covers our data collection period (winter 2020 and late spring 2021). Moreover, the pandemic surge corresponding to this period was milder in Spain compared to the UK. This brought about a certain sense of “fighting the virus alone” that may explain why frontline HCWs are seen by their colleagues as exhausted and psychologically distressed 1 year after the first pandemic surge. According to one of our KI interviewees, as compared to the first wave, where the country was under strict lockdown and signs of support for HCWs were shown every day, “the hardest thing during the second and third waves was to see people crowding in bars while you had to go to war every day.”

### Mental causal models supplement empirical causal models

During the KI interviews, we asked our participants about the causes and determinants of HCWs' mental health problems. Our main finding is that the perceived (i.e., subjective) causal mechanisms work at multiple levels –and, importantly, many of these subjective causal models align with epidemiological studies. This finding is in line with the multilevel models in psychiatric epidemiology, which argue that researchers exploring the determinants of mental health problems must analyse not only individual-level variables but also potential causes at higher levels (e.g., interpersonal, community, region) (50). Further, this has important implications for knowledge transfer activities (41) because it targets decision-makers at different levels (e.g., work, community, state). At the *intrapersonal level*, our primary finding was that some factors mediate exposures, such as spending time with COVID-19 patients, sharing a household with vulnerable people, or lack of scientific evidence to treat the infection and outcomes, i.e., health problems related to mental illness. From a transactional perspective, this result has a major implication. Even when the stressor is unmodifiable (e.g., living with an 80-year-old relative or vaccines not yet developed), psychological strategies can still modify mediational factors, such as fears or appraisal styles (51, 52). At the *interpersonal level*, HCWs reported determinants across various problems, which is also in line with COVID-19 studies conducted among HCWs (53, 54). Importantly, we found that perceived social isolation is a consequence of many reported problems. During the initial pandemic outbreak, HCWs in Spain were not only highly distressed and worried about infecting their loved ones; some decided to isolate themselves to protect them both physically and emotionally. Preventing HCWs from feeling alone may help reduce the negative consequences of mental health problems while increasing a key protective factor, namely social support. Importantly, reducing the so-called social support barriers (i.e., factors that reduce the use of social support, even when available) might also help improve HCWs' mental health (55). At the *organizational level*, HCWs reported common causes across problems: increased workload, shortages of protective equipment, or lack of standardized operating procedures. HCWs' mental models are thus in line with causal knowledge from epidemiological studies showing that crucial pandemic-related factors, such as having access to protective equipment or not having clear indications on how to prioritize access to mechanical ventilation, are associated with adverse mental health outcomes (21, 24). At the *community level*, our major finding was that HCW did not report that the wider community and people in it had a negative impact on their mental health –only one interviewee mentioned not feeling supported by other people during the second and the third pandemic waves. In Spain, widespread signs of support for HCWs were displayed every day during the first half of 2020 (the so-called “*aplauso sanitario*” or “clapping hands”), contrary to other COVID-19 hotspots around the globe (56, 57). This support can be seen as a spontaneous anti-stigma campaign that may have protected HCWs, who are often discriminated against and even attacked in other pandemic hotspots (56–59). With mounting evidence suggesting an association between reported discrimination and poor mental health outcomes among HCWs (60–62), mental health strategies at the community level could take the form of anti-stigma campaigns. Last, we found that HCWs rarely refer to determinants at the *public policy level*. The main reason is that causes described at all levels, such as increased exposure to SARS-CoV-2 (intrapersonal), the vulnerability of relatives and friends (interpersonal), shortage of protective equipment (organizational), or reduced scientific knowledge about SARS-CoV-2 (community), could be all seen as public policy issues. We described them as part of the “lower” levels because that helps tailor strategies and tackle decision-makers on the field (e.g., PPE shortage is a public health issue, but organization leaders might prevent or fix it). Moreover, while we adopted a multilevel approach when analyzing the data, we did not modify the study design, and participants were not probed for providing answers across levels.

### Frequently overlooked mental health factors can inform mental health programs

We used open-ended instead of close questions during FL interviews to capture as many problems as possible without any aprioristic constraints. We found that HCWs reported internalizing symptoms such as anxiety and depression quite frequently, roughly in line with the surveys used in prior studies (3, 63). However, they also mentioned externalizing symptoms, such as anger or hostility, and transdiagnostic symptoms, such as worry, guilt, or intolerance of uncertainty. Further, we also found that most problems and their determinants and consequences were interconnected (e.g., psychological distress was one of the causes of interpersonal conflicts), in line with previous studies using the same research design (46, 64). These findings support the design of mental health programs tailored for HCWs that target psychological distress in general –instead of specific mental health syndromes. Scalable transdiagnostic interventions have proven effective before in global settings (65, 66) and during the COVID-19 pandemic (67), although they away rigorous testing among care home workers during the COVID-19 pandemic (68). The question of whether it might help HCWs during the COVID-19 pandemic, if contextually adapted, remains unanswered.

### Some needs remain unmet

One promising finding was that HCWs reported strategies being implemented at all levels to face mental health problems, mostly psychological distress and fear of infection. Importantly, even when most professional psychological support, both for HCWs and other mental health patients, was provided through phone or video calls (69), our informants said they used it to reduce their psychological distress, stress, and moral injury. Notwithstanding, most of these strategies were implemented at the intrapersonal, interpersonal, and community levels but not at the organizational or public policy levels. For instance, regarding psychological distress, our respondents reported that, whereas frontline workers used self-help techniques, sought peer support, or started psychotherapy, they did not perceive that the organization leaders or policymakers ensured information flow, promoted occupational health, hired mental health specialists, or improved working conditions. This inaction brought a widespread sense of disbelief among HCWs, who thought they were running the extra mile but did not see their needs adequately covered.

### Limitations

We acknowledge the following limitations. First, we asked FL interviewees to report problems faced by frontline HCWs *since the beginning of the pandemic* without distinguishing between current and previous concerns, limiting our capacity to inform actions aimed at ameliorating ongoing problems rapidly. Second, we had to keep KI interviews as short as possible due to time and social distancing constraints. Following the research protocol, we collapsed some of the problems reported by FL interviewees, which allowed us to inquire about more problems in our KI interviews but increased the risk of losing nuances for a more fine-grained analysis –especially in heterogeneous problems like psychological distress. Third, and relatedly, we had to set aside the focus groups included in the DIME protocol due to the pressuring job duties during the third pandemic wave and the social distancing measures. Last, we followed the DIME protocol and did not probe KI interviewees for causes or strategies across levels (e.g., “*in their organization*, what do frontline HCWs generally perceive as causes of PROBLEM X?”). We may have thus missed potential determinants and strategies that are less accessible by open-ended questions, especially at the organizational and public policy levels.

## Conclusion

Over the years, globalization has increased the transmission rates of diseases worldwide (70), and as the world gets more globalized, likely, future pandemics will travel faster. As of January 1st, 2021 (when data was collected), Spain showed one of the highest excess mortality rates in Europe (16%), similar to that of Italy, the USA, or Brazil (12) –a finding particularly shocking for a country with one of the best-rated national health systems in the world (71). Although our findings reflect HCWs' perceptions of problems and needs –which may not match empirical findings, these subjective views can help inform research, policy, and practice to prepare health systems for future pandemics. In terms of research, we need the high-quality data available to prepare evidence-based public health strategies. A good balance between rapid and in-depth appraisals and between qualitative and quantitative methods is warranted. Regarding policy, we found that most reported causes and problems are modifiable, yet HCWs see them as not being implemented. If put in place earlier, preventative mental health strategies may help ameliorate the acute mental health impact and its mid-and long-term effects on HCWs and health systems in the future. Our findings suggest that such strategies could be set up at all levels, from intrapersonal to public health. At the individual level, self-help strategies are already being used and might help with various mental health problems. At the interpersonal level, informal peer support is seen by most HCWs as very useful for overcoming difficult working conditions. At the organization level, our informants call for actions that promote collaborative work, allow reassignments when needed, build strong leadership, or promote a (mentally) healthy working environment. At the community level, anti-stigma campaigns might be good to reduce the sense of loneliness and exhaustion frequently reported after the initial pandemic outbreak. Last, at the public policy level, offering specific mental health support and improving working conditions might also help with several mental health problems. In terms of practice, our findings may help design, adapt, and implement transdiagnostic mental health programs tailored for HCWs that can be rapidly implemented and scaled up from the early moments of future health crises. Notably, such programs might rely on already available resources, such as peer support or self-help, always following the restrictive measures to contain epidemics. In Spain, therefore, the RESPOND consortium will explore the effectiveness of a stepped-care program of scalable, internet-based psychological interventions locally adapted for HCWs working in an early pandemic hotspot.

## Data availability statement

The datasets presented in this article are not readily available because the qualitative data is not anonymized and, therefore, can not be shared. Requests to access the datasets should be directed to KM, kerry.rodriguez@uam.es.

## Ethics statement

The studies involving human participants were reviewed and approved by Hospital La Paz in Madrid (study ID: 4498) and Parc Sanitari Sant Joan de Déu in Barcelona (study ID: PIC-277-20). The patients/participants provided their written informed consent to participate in this study.

## The RESPOND consortium

The RESPOND Consortium includes the following collaborators: Giovanni Corrao, Josep Maria Haro, Antonio Lora, Raffael Kalisch, Vincent Lorant, Ellenor Mittendorfer-Rutz, Brenda Penninx, Henrik Walter, and Rinske Roos.

## Author contributions

Investigation and project administration: RM, KM, MF-N, AM-M, M-FB-O, CB, BR-V, and JA-M. Writing—original draft: RM and KM. Supervision: JA-M. Funding acquisition: PN, MS, MP, MM, DM, RB, and JA-M. Conceptualization, methodology, data curation, and writing—review and editing: All authors. All authors contributed to the article and approved the submitted version.

## Funding

The RESPOND project was funded under Horizon 2020—the Framework Programme for Research and Innovation (2014–2020) (grant number: 101016127) and the work of MF-N was supported by a postdoctoral fellowship of the ISCIII (CD20/00036).

## Conflict of interest

The authors declare that the research was conducted in the absence of any commercial or financial relationships that could be construed as a potential conflict of interest.

## Publisher's note

All claims expressed in this article are solely those of the authors and do not necessarily represent those of their affiliated organizations, or those of the publisher, the editors and the reviewers. Any product that may be evaluated in this article, or claim that may be made by its manufacturer, is not guaranteed or endorsed by the publisher.

## Author disclaimer

The content of this article reflects only the authors' views, and the European Community is not liable for any use that may be made of the information contained therein.

## References

[1] Aymerich C Pedruzo B Pérez JL Laborda M Herrero J Blanco J. COVID-19 pandemic effects on health worker's mental health: Systematic review and meta-analysis. Eur Psychiatry. (2022) 65:e10. 10.1192/j.eurpsy.2022.1 35060458 PMC8828390

[2] Johns G Samuel V Freemantle L Lewis J Waddington L. The global prevalence of depression and anxiety among doctors during the covid-19 pandemic: Systematic review and meta-analysis. J Affect Disord. (2022) 298:431–41. 10.1016/j.jad.2021.11.026 34785264 PMC8596335

[3] Pappa S Ntella V Giannakas T Giannakoulis VG Papoutsi E Katsaounou P. Prevalence of depression, anxiety, and insomnia among healthcare workers during the COVID-19 pandemic: A systematic review and meta-analysis. Brain Behav Immun. (2020) 88:901–7. 10.1016/j.bbi.2020.05.026 32437915 PMC7206431

[4] Alimoradi Z Ohayon MM Griffiths MD Lin CY Pakpour AH. Fear of COVID-19 and its association with mental health-related factors: systematic review and meta-analysis. BJ Psych Open. (2022) 8:26. 10.1192/bjo.2022.26 35307051 PMC8943231

[5] Alimoradi Z Broström A Tsang HWH Griffiths MD Haghayegh S Ohayon MM. Sleep problems during COVID-19 pandemic and its' association to psychological distress: a systematic review and meta-analysis. EClinicalMedicine. (2021) 36:100916. 10.1016/j.eclinm.2021.100916 34131640 PMC8192091

[6] World Health Organization. Mental Health and COVID-19: Early Evidence of the Pandemic's Impact. Geneva: World Health Organization (2022)

[7] Alonso J Vilagut G Alayo I Ferrer M Amigo F Aragón-Peña A. Mental impact of Covid-19 among Spanish healthcare workers. A large longitudinal survey. Epidemiol Psychiatr Sci. (2022) 31:e28. 10.1017/S2045796022000130 35485802 PMC9069586

[8] Canal-Rivero M Armesto-Luque L Rubio-García A Rodriguez-Menéndez G Garrido-Torres N Capitán L. Trauma and stressor-related disorders among health care workers during COVID-19 pandemic and the role of the gender: a prospective longitudinal survey. J Affect Disord. (2022) 302:110–22. 10.1016/j.jad.2022.01.021 35032507 PMC8755453

[9] Mediavilla R Fernandez-Jimenez E Martinez-Morata I Jaramillo F Andreo-Jover J Moran-Sanchez I. Sustained Negative Mental Health Outcomes Among Healthcare Workers Over the First Year of the COVID-19 Pandemic: A Prospective Cohort Study. (2021). Available online at: https://www.medrxiv.org/content/10.1101/2021.11.21.21266594v1 (accessed 26 de noviembre de 2021) 10.3389/ijph.2022.1604553 PMC9266625 35814735

[10] Sasaki N Asaoka H Kuroda R Tsuno K Imamura K Kawakami N. Sustained poor mental health among healthcare workers in COVID-19 pandemic: a longitudinal analysis of the four-wave panel survey over 8 months in Japan. J Occup Health enero de. (2021) 63:e12227. 10.1002/1348-9585.12227 34021683 PMC8140377

[11] Borek AJ Pilbeam C Mableson H Wanat M Atkinson P Sheard S. Experiences and concerns of health workers throughout the first year of the COVID-19 pandemic in the UK: a longitudinal qualitative interview study. PLOS ONE. (2022) 17:e0264906. 10.1371/journal.pone.0264906 35294450 PMC8926177

[12] Ritchie H Mathieu E Rodés-Guirao L Appel C Giattino C Ortiz-Ospina E. Coronavirus Pandemic (COVID-19). Available online at: https://ourworldindata.org/covid-cases.

[13] Schug C Geiser F Hiebel N Beschoner P Jerg-Bretzke L Albus C. Sick leave and intention to quit the job among nursing staff in German hospitals during the COVID-19 pandemic. Int J Environ Res Public Health. (2022) 19:1947. 10.3390/ijerph19041947 35206136 PMC8872054

[14] Stefanovska - Petkovska M Stefanovska VV Bojadjieva S Bojadjiev MI. Psychological distress, burnout, job satisfaction and intention to quit among primary healthcare nurses. Health Serv Manage Res. (2021) 34:92–8. 10.1177/0951484820971444 33156712

[15] De los Santos JAA Labrague LJ. The impact of fear of COVID-19 on job stress, and turnover intentions of frontline nurses in the community: a cross-sectional study in the Philippines. Traumatology. (2021) 27:52–9. 10.1037/trm0000294

[16] Instituto de Salud Carlos III. Vigilancia de los excesos de mortalidad por todas las causas. Madrid: Instituto de Salud Carlos III (2020)

[17] Instituto de Salud Carlos III. Informe n^o^ 19. Situación de COVID-19. Madrid: Instituto de Salud Carlos III (2020).

[18] Instituto de Salud Carlos III. COVID-19. Madrid: Instituto de Salud Carlos III (2020).

[19] Martínez-Alés G Domingo-Relloso A Arribas JR Quintana-Díaz M Hernán MA. the COVID@HULP Group. Critical care requirements under uncontrolled transmission of SARS-CoV-2. Am J Public Health. (2021) 111:923–6. 10.2105/AJPH.2020.306151 33734835 PMC8034012

[20] World Health Organization International Labour Organization. Caring for Those who Care: National Programmes for Occupational Health for Health Workers. Policy brief. Geneva: World Health Organization (2020).

[21] Alonso J Vilagut G Mortier P Ferrer M Alayo I Aragón-Peña A. Mental health impact of the first wave of COVID-19 pandemic on Spanish healthcare workers: a large cross-sectional survey. Revista de Psiquiatrí*a y Salud Mental*. (2021) 14:90–105. 10.1016/j.rpsm.2020.12.001 34127211 PMC10068024

[22] Kunzler AM Röthke N Günthner L Stoffers-Winterling J Tüscher O Coenen M. Mental burden and its risk and protective factors during the early phase of the SARS-CoV-2 pandemic: systematic review and meta-analyses. Global Health 29. (2021) 17:34. 10.1186/s12992-021-00670-y 33781283 PMC8006628

[23] Lasalvia A Bonetto C Porru S Carta A Tardivo S Bovo C. Psychological impact of COVID-19 pandemic on healthcare workers in a highly burdened area of north-east Italy. Epidemiol Psychiatr Sci. (2021) 17:e1. 10.1017/S2045796020001158 33331255 PMC7804082

[24] Mediavilla R Fernández-Jiménez E Martínez-Alés G Moreno-Küstner B Martínez-Morata I Jaramillo F. Role of access to personal protective equipment, treatment prioritization decisions, and changes in job functions on health workers' mental health outcomes during the initial outbreak of the COVID-19 pandemic. J Affect Disord. (2021) 295:405–9. 10.1016/j.jad.2021.08.059 34507219 PMC8403068

[25] Morawa E Schug C Geiser F Beschoner P Jerg-Bretzke L Albus C. Psychosocial burden and working conditions during the COVID-19 pandemic in Germany: the VOICE survey among 3678 health care workers in hospitals. J Psychosom Res. (2021) 144:110415. 10.1016/j.jpsychores.2021.110415 33743398 PMC7944879

[26] Olashore AA Akanni OO Fela-Thomas AL Khutsafalo K. The psychological impact of COVID-19 on health-care workers in African Countries: a systematic review. Asian J Soc Health Behav. (2021) 4:85. 10.4103/shb.shb_32_21

[27] Priede A López-Álvarez I Carracedo-Sanchidrián D González-Blanch C. Mental health interventions for healthcare workers during the first wave of COVID-19 pandemic in Spain. Rev Psiquiatr Salud Ment (Engl Ed). (2021) 14:83–9. 10.1016/j.rpsm.2021.01.005 34127210 PMC8194007

[28] Rodriguez-Vega B Palao Á Muñoz-Sanjose A Torrijos M Aguirre P Fernández A. Implementation of a mindfulness-based crisis intervention for frontline healthcare workers during the COVID-19 outbreak in a public general hospital in Madrid, Spain. Front Psychiatry. (2020) 11:562578. 10.3389/fpsyt.2020.562578 33329103 PMC7673433

[29] Bai Y Lin CC Lin CY Chen JY Chue CM Chou P. Survey of stress reactions among health care workers involved with the SARS outbreak. PS. (2004) 55:1055–7. 10.1176/appi.ps.55.9.1055 15345768

[30] Lee SM Kang WS Cho AR Kim T Park JK. Psychological impact of the 2015 MERS outbreak on hospital workers and quarantined hemodialysis patients. Compr Psychiatry. (2018) 87:123–7. 10.1016/j.comppsych.2018.10.003 30343247 PMC7094631

[31] Wilder-Smith A Chiew CJ Lee VJ. Can we contain the COVID-19 outbreak with the same measures as for SARS? Lancet Infect Dis. (2020) 20:e102–7. 10.1016/S1473-3099(20)30129-8 32145768 PMC7102636

[32] Telenti A Arvin A Corey L Corti D Diamond MS García-Sastre A. After the pandemic: perspectives on the future trajectory of COVID-19. Nature. (2021) 596:495–504. 10.1038/s41586-021-03792-w 34237771

[33] Salazar de Pablo G Vaquerizo-Serrano J Catalan A Arango C Moreno C Ferre F. Impact of coronavirus syndromes on physical and mental health of health care workers: systematic review and meta-analysis. J Affect Disord. (2020) 275:48–57. 10.1016/j.jad.2020.06.022 32658823 PMC7314697

[34] Palinkas LA. Qualitative and mixed methods in mental health services and implementation research. J Clin Child Adolesc Psychol. (2014) 43:851–61. 10.1080/15374416.2014.910791 25350675 PMC4212209

[35] Ayuso-Mateos JL Miret M Lopez-Garcia P Alem A Chisholm D Gureje O. Effective methods for knowledge transfer to strengthen mental health systems in low- and middle-income countries. BJ Psych Open. (2019) 5:50. 10.1192/bjo.2019.50 31530323 PMC6688465

[36] Moore GF Audrey S Barker M Bond L Bonell C Hardeman W. Process evaluation of complex interventions: medical research council guidance. BMJ. (2015) 350:h1258. 10.1136/bmj.h1258 25791983 PMC4366184

[37] Brownson RC Fielding JE Maylahn CM. Evidence-Based public health: a fundamental concept for public health practice. Annu Rev Public Health. (2009) 30:175–201. 10.1146/annurev.publhealth.031308.100134 19296775

[38] Applied Mental Health Research Group. Design, Implementation, Monitoring, and Evaluation of mental health and psychosocial assistance programs for trauma survivors in low resource countries: A user's manual for researchers and program implementers (adult version). Baltimore, USA: John Hopkins University (2013). p. 66

[39] Annells M. Grounded theory method: philosophical perspectives, paradigm of inquiry, and postmodernism. Qual Health Res. (1996) 6:379–93. 10.1177/104973239600600306

[40] Braun V Clarke V. Using thematic analysis in psychology. Qual Res Psychol. (2006) 3:77–101. 10.1191/1478088706qp063oa

[41] McLeroy KR Bibeau D Steckler A Glanz K. An ecological perspective on health promotion programs. Health Educ Q. (1988) 15:351–77. 10.1177/109019818801500401 3068205

[42] Wallace JE. Mental health and stigma in the medical profession. Health (London). (2012) 16:3–18. 10.1177/1363459310371080 21177717

[43] Hennein R Lowe S. A hybrid inductive-abductive analysis of health workers' experiences and wellbeing during the COVID-19 pandemic in the United States. PLoS ONE. (2020) 15:e0240646. 10.1371/journal.pone.0240646 33104711 PMC7588050

[44] Brown FL Aoun M Taha K Steen F Hansen P Bird M. The cultural and contextual adaptation process of an intervention to reduce psychological distress in young adolescents living in Lebanon. Front Psychiatry. (2020) 11:212. 10.3389/fpsyt.2020.00212 32265759 PMC7104812

[45] Burchert S Alkneme MS Bird M Carswell K Cuijpers P Hansen P. User-Centered app adaptation of a low-intensity e-mental health intervention for Syrian refugees. Front Psychiatry. (2019) 9:663. 10.3389/fpsyt.2018.00663 30740065 PMC6355704

[46] Chiumento A Rutayisire T Sarabwe E Hasan MT Kasujja R Nabirinde R. Exploring the mental health and psychosocial problems of Congolese refugees living in refugee settings in Rwanda and Uganda: a rapid qualitative study. Confl Health. (2020) 14:77. 10.1186/s13031-020-00323-8 33292363 PMC7670672

[47] López Steinmetz LC Herrera CR Fong SB Godoy JC. A Longitudinal study on the changes in mental health of healthcare workers during the COVID-19 pandemic. Psychiatry. (2021) 85:56–71. 10.1080/00332747.2021.1940469 34328825

[48] Pinho L Correia T Sampaio F Sequeira C Teixeira L Lopes M. The use of mental health promotion strategies by nurses to reduce anxiety, stress, and depression during the COVID-19 outbreak: a prospective cohort study. Environ Res. (2021) 195:110828. 10.1016/j.envres.2021.110828 33548294 PMC7857980

[49] Organización Colegial de Enfermería. Radiografía de la situación profesional y emocional de la profesión enfermera. Madrid: CGE (2022). p. 41.

[50] Schwartz S Susser E Susser M. A Future for epidemiology? Annu Rev Public Health. (1999) 20:15–33. 10.1146/annurev.publhealth.20.1.15 10352847

[51] Lazarus RS Folkman S. Stress, Appraisal, and Coping. Springer Publishing Company (1984). p. 460.

[52] Lazarus RS Folkman S. Transactional theory and research on emotions and coping. Eur J Pers. (1987) 1:141–69. 10.1002/per.2410010304

[53] Magnavita N Soave PM Antonelli M. Prolonged stress causes depression in frontline workers facing the COVID-19 pandemic—a repeated cross-sectional study in a covid-19 hub-hospital in central Italy. Int J Environ Res Public Health. (2021) 18:7316. 10.3390/ijerph18147316 34299767 PMC8304927

[54] Ortiz-Calvo E Martínez-Alés G Mediavilla R González-Gómez E Fernández-Jiménez E Bravo-Ortiz MF. The role of social support and resilience in the mental health impact of the COVID-19 pandemic among healthcare workers in Spain. J Psychiatr Res. (2021) 148:181–7. 10.1016/j.jpsychires.2021.12.030 35124398 PMC8668396

[55] Thoresen S Jensen TK Wentzel-Larsen T Dyb G. Social support barriers and mental health in terrorist attack survivors. J Affect Disord. (2014) 156:187–93. 10.1016/j.jad.2013.12.014 24398044

[56] Devi S. COVID-19 exacerbates violence against health workers. Lancet. (2020) 396:658. 10.1016/S0140-6736(20)31858-4 32891198 PMC7470723

[57] Taylor S Landry CA Rachor GS Paluszek MM Asmundson GJG. Fear and avoidance of healthcare workers: an important, under-recognized form of stigmatization during the COVID-19 pandemic. J Anxiety Disord. (2020) 75:102289. 10.1016/j.janxdis.2020.102289 32853884 PMC7434636

[58] McKay D Heisler M Mishori R Catton H Kloiber O. Attacks against health-care personnel must stop, especially as the world fights COVID-19. Lancet. (2020) 395:1743–5. 10.1016/S0140-6736(20)31191-0 32445692 PMC7239629

[59] Patel BR Khanpara BG Mehta PI Patel KD Marvania NP. Evaluation of perceived social stigma and burnout, among health-care workers working in covid-19 designated hospital of India: a cross-sectional study. Asian J Soc Health Behav. (2021) 4:156. 10.4103/shb.shb_54_21

[60] Elhadi M Msherghi A Elgzairi M Alhashimi A Bouhuwaish A Biala M. Psychological status of healthcare workers during the civil war and COVID-19 pandemic: a cross-sectional study. J Psychosom Res. (2020) 137:110221. 10.1016/j.jpsychores.2020.110221 32827801 PMC7428743

[61] Mediavilla R Fernández-Jiménez E Andreo J Morán-Sánchez I Muñoz-Sanjosé A Moreno-Küstner B. Association Between Perceived Discrimination and Mental Health Outcomes Among Health Workers During the Initial COVID-19 outbreak. Available online at: https://www.sciencedirect.com/science/article/pii/S1888989121000628 (accessed July 04, 2022). 10.1016/j.rpsm.2021.06.001 PMC8253602 34153496

[62] Ramaci T Barattucci M Ledda C Rapisarda V. Social Stigma during COVID-19 and its Impact on HCWs Outcomes. Sustainability. (2020) 12:3834. 10.3390/su1209383434993856

[63] Santabárbara J Bueno-Notivol J Lipnicki DM Olaya B Pérez-Moreno M Gracia-García P. Prevalence of anxiety in health care professionals during the COVID-19 pandemic: a rapid systematic review (on published articles in Medline) with meta-analysis. Prog Neuropsychopharmacol Biol Psychiatry. (2021) 107:110244. 10.1016/j.pnpbp.2021.110244 33453320 PMC9188432

[64] Lee C Nguyen AJ Russell T Aules Y Bolton P. Mental health and psychosocial problems among conflict-affected children in Kachin State, Myanmar: a qualitative study. Confl Health. (2018) 12:39. 10.1186/s13031-018-0175-8 30250500 PMC6145186

[65] Rahman A Khan MN Hamdani SU Chiumento A Akhtar P Nazir H. Effectiveness of a brief group psychological intervention for women in a post-conflict setting in Pakistan: a single-blind, cluster, randomised controlled trial. Lancet. (2019) 393:1733–44. 10.1016/S0140-6736(18)32343-2 30948286

[66] Tol WA Leku MR Lakin DP Carswell K Augustinavicius J Adaku A. Guided self-help to reduce psychological distress in South Sudanese female refugees in Uganda: a cluster randomised trial. The Lancet Global Health 1. (2020) 8:e254–63. 10.1016/S2214-109X(19)30504-2 31981556 PMC9899129

[67] Bryant RA Dawson KS Keyan D Azevedo S Yadav S Tran J. Effectiveness of a Videoconferencing-Delivered Psychological Intervention for Mental Health Problems during COVID-19: A Proof-of-Concept Randomized Clinical Trial. Psychother Psychosom. (2021) 7:1–10. 10.1159/000520283 34875669 PMC8820421

[68] Riello M Purgato M Bove C Tedeschi F MacTaggart D Barbui C. Effectiveness of self-help plus (SH+) in reducing anxiety and post-traumatic symptomatology among care home workers during the COVID-19 pandemic: a randomized controlled trial. R Soc Open Sci. (2021) 8:210219. 10.1098/rsos.210219 34849238 PMC8611343

[69] Mediavilla R Fernández-Jiménez E Rodríguez-Vega B Gotor-Martínez L Rivelles-Sevilla RV Rojano-Capilla P. Adapting mental health care after the COVID-19 outbreak: preliminary findings from a public general hospital in Madrid (Spain). Psychiatr Res. (2020) 12:113077. 10.1016/j.psychres.2020.113077 32434094 PMC7214322

[70] Shrestha N Shad MY Ulvi O Khan MH Karamehic-Muratovic A Nguyen USDT. The impact of COVID-19 on globalization. One Health. (2020) 11:100180. 10.1016/j.onehlt.2020.100180 33072836 PMC7553059

[71] Barber RM Fullman N Sorensen RJD Bollyky T McKee M Nolte E. Healthcare Access and Quality Index based on mortality from causes amenable to personal health care in 195 countries and territories, 1990–2015: a novel analysis from the global burden of disease study 2015. Lancet. (2017) 15:390. 10.1016/S0140-6736(17)30818-8 28528753 PMC5528124

