# Supplementary material for: Mental health problems and needs of frontline healthcare workers during the COVID-19 pandemic in Spain: A qualitative analysis
Source: Front Public Health. 2022 Jul 27;10:956403. doi: 10.3389/fpubh.2022.956403 (PMC9363705; doi:10.3389/fpubh.2022.956403)
Supplement: Supplementary file 1 [file Data_Sheet_1.docx]

***Supplementary Material***

1. **Supplementary Tables**

**Supplementary Table 1.** Key informants’ quotes support subjective models of mental health models (the numbers reflect the associations shown in Figure 1).

| **Problem** | **#** | **ID** | **Quote (SPA/CAT)** | **Quote (ENG)** |
| --- | --- | --- | --- | --- |
| PsyDis | 1 | HULP50 | "No hemos tenido tiempo o no se nos ha permitido desconectar en ningún momento o desactivarnos. Y seguimos en esa línea. Entonces al final las activaciones mantenidas en el tiempo lo que hacen es quemar el sistema" | “We haven’t had the time or haven’t been able to disconnect or reduce the activation level, and we are carrying on like this. In the end, the activation maintained over time will burn the system” |
| PsyDis | 1 | HULP47 | "No podía dormir por la noche porque me venían a la cabeza las imágenes que había vivido durante todo el día" | “I couldn't sleep at night cause images of what I experienced during the day came to my mind” |
| PsyDis | 1 | HULP47 | "Nos ha pillado sin herramientas psicológicas para tratar de sobrellevarlo mejor" | "It has caught us without psychological skills to try to cope with it better" |
| PsyDis | 1 | HULP51 | "La gente activa, deportista, lo ha pasado peor porque no podía hacer nada (…) Ha sido todo muy pasivo" | "Active, athletic people have had a harder time because they couldn't do anything. Everything has been very passive" |
| PsyDis | 1 | HULP44 | "Había plantas donde morían todos. Esa gente lo pasó fatal" | “In some wards, everyone died. Those people had a hard time” |
| PsyDis | 1 | HULP47 | "Si alguien iba a morir le acompañábamos, porque morían solos, sin sus familiares" | "If someone was going to die, we accompanied them because they died alone, without their relatives" |
| PsyDis | 2 | HULP47 | "He visto que los compañeros que se han aislado totalmente de sus familias (…) lo han pasado peor" | “I’ve seen that it’s been harder for workmates that have completely isolated themselves from their families” |
| PsyDis | 2 | HULP47 | "Al principio sí intenté aislarme en mi casa porque tenía miedo de contagiar [a mi familia] (…) luego (…) sentí que si no estaba con ellos no iba a poder aguantar aquella presión del trabajo" | In the beginning, I did try to isolate myself in my house because I was afraid of infecting [my family]; later (…) I felt that if I weren’t with them, I wouldn’t be able to stand the work pressure. |
| PsyDis | 2 | HULP49 | "Nos han quitado muchas cosas que nos hacían sentir bien: vida social, viajes, vacaciones" | They have taken away many things that made us feel good: social life, trips, holidays. |
| PsyDis | 2 | HULP51 | "Cuando tienes un mal día de trabajo, me voy a aprovechar la tarde, pero no se podía aprovechar la tarde si estás encerrada" | When I had a bad day at work, I could enjoy the afternoon, but you couldn't take advantage of the afternoon if you were locked up. |
| PsyDis | 3 | HULP47 | "No llegabas a tiempo a todos los pacientes" | “There wasn't enough time to (be able to) attend adequately to all patients” |
| PsyDis | 3 | HULP47 | "Sentía mucha impotencia de no poder atender a la gente dignamente" | I felt very helpless, not being able to take care of people decently. |
| PsyDis | 4 | PSSJD_07 | “Buscas información y te encuentras tanta información en tan poco tiempo que a veces se contradecía (...) lo que estábamos pensando que hacíamos bien después resulta que no era evidencia científica o que se ha demostrado en los estudios posteriores que no" | “You look for information and find so much information in little time that sometimes it contradicts itself; What we thought we were doing well, we then found out it wasn’t scientific evidence, or that further studies had proven otherwise” |
| PsyDis | 5 | PSSJD_01 | "A mi m'aniria bé per exemple un grup de treball o un equip (...) on compartir les seves experiències i sentir recolzat i qui no està sol" | "I would do well with, for example, a working group or a team (...) where you can share your experiences and feel supported and that you are not alone." |
| PsyDis | 6 | HULP46 | "Lo difícil de las olas [segunda y tercera] es que tú seguías viniendo a la guerra y sabías que la terraza de enfrente ya estaba llena de gente. Y hay gente que dice 'pero si lo estoy pasando mal para nada” | “The hard part of the waves [second and third] is that you continued to go to war, and you knew that the bar terrace in front of you was full of people. Some people said they were having a bad time, but it wasn't the case” |
| FoF | 7 | HULP49 | "Tenía mucho menos miedo en la primera ola porque no estaba con pacientes COVID (…) pero ahora [veo] gente todos los días que se está muriendo y a sus familias echas polvo y (…) crees que te va a pasar a ti" | "I was a lot less afraid in the first wave cause I wasn't with COVID patients (...), but now, I see people dying every day and their torn families, and (...) you can't help thinking that it's going to happen to you too" |
| FoF | 8 | HULP49 | "En esta segunda y en adelante olas sí que he tenido bastante miedo porque mi chica está embarazada. Entonces ahí sí ha sido un poco agobiante (…) la sensación. Y porque he estado mucho más en contacto con el virus. Entonces pues miedo. Cada vez que oyes a alguien toser, tanto (...) en el tren como pacientes COVID, he tenido miedo a contagiarme" | “I was terrified in the first and second waves because my girlfriend was pregnant. At that moment, I was a bit overwhelmed by the feeling. Also, I had been much more in contact with the virus. Whenever I hear someone cough on the train or COVID patients, I am scared of getting infected” |
| FoF | 8 | HULP51 | "[Había] compañeros de trabajo que vivían con su madre de 90 años, con diabetes y cáncer, que no se quitaban la mascarilla ni para dormir" | "Some work colleagues lived with their elderly mother, who had diabetes and cancer, and they didn't remove their face mask, not even in bed." |
| FoF | 9 | HULP48 | "No había equipos de protección o no sabíamos cómo utilizarlos" | “We didn't have protective gear, or we didn't know how to use it” |
| FoF | 9 | HULP44 | "La mayoría iban con miedo por falta de equipo, no porque tengan miedo con los equipos puestos" | "Most of us were scared because of the lack of equipment, not because we were afraid with the equipment on " |
| FoF | 9 | HULP44 | "Había sensación de vulnerabilidad porque no teníamos equipos" | "There was a sense of vulnerability because we didn't have equipment" |
| FoF | 9 | PSSJD_02 | "Ese miedo por lo que yo he observado se ha explicitado en mucha angustia y (…) está asociado con mi creencia de que no teníamos epis adecuados ni materiales adecuados para para poder entrar en contacto con las personas COVID . | "The fear of what I’ve witnessed has brought a lot of distress to the surface, and I believe it’s related to the fact that we didn’t have adequate PPE or materials to face people with COVID" |
| FoF | 10 | HULP47 | "Había tanto trabajo que no podías ni ir al baño" | "There was so much work to do you couldn't even go to the bathroom” |
| FoF | 11 | HULP47 | "No se sabía cómo tratar a este tipo de pacientes, que eran muy delicados" | "We didn't know how to treat this kind of patient, which were so delicate" |
| FoF | 11 | HULP48 | "Estaba también el miedo a lo que iba a pasar, a lo desconocido, a no saber qué es lo que estabas viviendo" | “There was also fear of what would happen, to the unknown, to not knowing what you were going through” |
| Stress | 12 | PSSJD_06 | "Llavors crec que hi ha unes condicions estructurals dels hospitals i més en un moment de crisi, d'una precarització que porten una mica a, crec que l'estrès no és només un problema personal dels recursos " | “I think there are structural conditions in the hospitals, and even more so in a moment of crisis, a precariousness that leads to... I believe that stress is not only a personal problem of having resources” |
| Stress | 13 | PSSJD_03 | "Per què de cop teníem una avalancha de feina que no erem capaços d'absorbir . El primer mes i mig . Feia torns de dotze hores cada dia de dilluns a diumenge” | “Because all of a sudden, we had an avalanche of work that we could not assimilate. The first month and a half I had twelve-hour shifts every day from Monday to Sunday” |
| Stress | 14 | PSSJD_13 | "hay unos protocolos que al otro dia cambiaban, y a día y medio , algo normal eh y que al final es algo desconocido el pero al final mentalmente es una barbaridad" | “Some protocols changed from one day to the other, and every day and a half, which is normal, eh? Because at the end of the day, it’s unfamiliar, but in the end, mentally, it’s brutal” |
| Stress | 14 | PSSJD_14 | "Les causes del problema [estrés] sobretot en aquest cas és enfrontar-te amb una malaltia que és nova i que no tens uns protocols a seguir, et van donant protocols els quals van canviar constantment, la part sindròmica, de tractament i de tot va canviant a mesura que vas treballant, el que era vàlid al principi en 24 o 48 hores, una setmana o dos ja no és vàlid, llavors donava una sensació d'inseguretat (...), i estrés" | “What causes the problem [stress], especially in this case, is facing a novel disease and not having protocols to follow. They give you constantly changing protocols regarding syndromes and treatment; everything changes as you work. What is valid initially wasn't valid 24 o 48 hours later, 2 or 3 weeks later. This led to a feeling of insecurity and stress” |
| MorDis | 15 | PSSJD_06 | "El nivell de formació de coneixement i de reflexió sobre la pràctica crítica que hi ha, que jo conec per l'experiència a les àrees de crítics i hospitals en general, en termes ètics és molt baixa. Les formacions sanitàries tenim un coneixement d'ètica aplicada baixíssim, de forma que de sobte ens vam trobar parlant de aspectes de si una vida valia més que una altra, si podien entrar o no els familiars, un qüestionament que es vivia d'una forma molt negativa però crec que més enllà del problema crec que els efectes es vivien en aquest malestar moral" | The level of knowledge and thought regarding the practice of critical care is relatively low in ethical terms. I know this because of my experience in critical care units and hospitals. There is a low level of applied ethics in healthcare training programmes, so suddenly, we found ourselves discussing whether one life had more value than another and whether relatives could come in or not. This line of questioning was seen negatively, but beyond the problem, I believe that the effects were experienced as moral distress. |
| MorDis | 16 | PSSJD_07 | "Creo que aparece por nuestra necesidad como sanitarios de salvar vidas. Esa cosa inculcadas de yo puedo, yo te salvaré o yo lo voy a intentar . Cuando los recursos o el cansancio no te lo permiten, o no conocer a lo que te estás enfrentando no te ayuda, yo creo que todo viene desencadenado de “Yo puedo , yo quiero , yo lo haré , estoy aquí para eso”. | "It comes from our need as health care workers to save lives. That instilled thing: I can do it, and I will save you or try to. When the resources or exhaustion doesn’t allow it, or not knowing what you are dealing with doesn’t help. I think it all comes from “I can, I want to, I will, I am here for that” |
| MorDis | 17 | PSSJD_05 | "La naturaleza de esta enfermedad que ha sido tan horrible" | “The nature of this illness has been so terrible” |
| MorDis | 18 | PSSJD_14 | "No tenir una pauta clara d’actuació, moltes vegades és això, no tenir una pauta clara d'actuació o tenir-la i no estar-hi d'acord" | “Not having a precise protocol, often it’s just that, not having a clear protocol or having it but not approving it |
| MorDis | 19 | PSSJD_05 | "El hecho de conocer cómo funciona la enfermedad da mayor seguridad por lo tanto hay menos impotencia , eh? Porque ya vas haciendo las cosas mejor . Sabes lo que has de hacer con más seguridad. Es verdad que la enfermedad evoluciona de una manera muy rápida a veces y buf! te da una sorpresa y ves que un paciente fallece sin esperarlo. Pero creo que ya no se vive tanto desde la culpa , sino sabiendo que la enfermedad tiene este proceso" | "Knowing how the disease works gives you more confidence, so there is less helplessness, eh? You are more confident about what you have to do. Indeed, the illness evolves quickly sometimes and uff! It surprises you, and you watch a patient die without expecting it. But I think we don’t live as much from guilt anymore, knowing that the illness has this process” |
| IntCon | 20 | HULP49 | "Factores que te he comentado como el miedo al contagio, la incertidumbre, o cómo esté cada uno, afectan a las relaciones de trabajo" | "Factors that I have mentioned to you, like fear of infection, uncertainty, or how we are feeling, affect workplace relationships." |
| IntCon | 20 | HULP49 | "Estás tan al límite de malestar que cualquier cosa te molesta más que antes" | "You were so on edge and upset that anything annoyed you more than usual" |
| IntCon | 21 | HULP48 | "Había mucha tensión entre colectivos porque estaba desdibujado lo que hacía cada uno. Yo creo que había grados de exposición y eso genera rivalidades" | “There was a lot of tension between groups because the task boundaries were blurry. In my opinion, there were levels of exposure that created rivalries” |
| IntCon | 21 | HULP48 | "Cada uno se sentía más expuesto: en el contagio, en el nivel de responsabilidad, o en la toma de decisiones. | "Each one felt more exposed: to the infection, the level of responsibility or the decision making" |
| IntCon | 22 | HULP49 | "Puede haber un choque de criterios (…) No es que uno tenga razón y el otro no, pero en cuanto dos personas piensan diferente puede haber tensión" | “There can be conflicting criteria (...) It’s not that one is right and the other one wrong, but when two people think differently, there can be tension” |
| IntCon | 22 | HULP43 | "Si un anestesista no lleva las mismas pautas [que un intensivista], a lo mejor uno piensa que hay que intubar y otro no (…) Son ésas las cosas que les ponen nerviosos, porque piensan que no está bien hecho, que no es la forma de llevar al paciente" | “If an anaesthetist doesn’t follow the same guidelines [as an intensivist], perhaps one thinks that is best to intubate and the other one not (...) These things make them nervous because they believe that it’s not done correctly, that that is not the way you should treat a patient" |
| IntCon | 23 | HULP47 | "Hay veces que hay conflictos entre compañeros, que uno quiere hacer una cosa y otro otra, y en momentos de tensión en los que no hay comunicación entre nosotros, surgen conflictos, tensiones" | “Some times there are conflicts between workmates, where one wants to do one thing and the other one another, and in moments of tension where there is a lack of communication among us, conflicts and tensions arise” |
| IntCon | 24 | HULP48 | "[La pandemia] ha destapado tensiones que antes estaban camufladas o toleradas y que, en un momento de elevada tensión y emocionalidad, ya no toleras. Dejas de tolerar lo que antes has tolerado" | "[The pandemic] has uncovered tensions that used to be hidden or tolerated, which, in a moment of heightened tension and emotion, you don't handle. You stop tolerating what you used to tolerate" |
| IntCon | 25 | HULP48 | "[Había distintos niveles de] implicación: gente que trabajaba más, gente que trabajaba menos, gente que se responsabiliza más, gente que se responsabiliza menos (…) Y eso producía diferencias y tensiones" | "[There were different levels of ] involvement: people who worked more, people who worked less, people who assumed more responsibility, people who assumed less responsibility (...) And this created differences and tensions" |
| IntCon | 26 | HULP48 | "Algún compañero se cabreaba con la supervisora, que no nos daba los EPI" | "Some workmates got mad with the supervisor, who didn't give us PPE" |
| IntCon | 27 | HULP51 | "Se nos ha pedido hacer cosas que no estábamos preparados para hacer" | "We have been asked to do things for which we weren't prepared to do" |
| IntCon | 27 | HULP46 | "Cada vez que abríamos una [nueva unidad] COVID parte del personal cambiaba y los equipos se estructuraban y reestructuraban en dos meses (…) Había falta de pertenencia a la unidad" | "Every time we opened a new COVID one [new unit], part of the staff changed, and teams were organised and reorganised in two months (...) There was a lack of belonging in the unit" |

*Note.* PsyDis = Psychological Distress, FoF = Fear of Infection, MorDis = Moral Distress, IntCon = Interpersonal Conflicts.

**Supplementary Table 2.** Sociodemographic Characteristics of Key informant (KI) interviewee.

| **ID** | **Site** | **Age group** | **Gender** | **Job** |
| --- | --- | --- | --- | --- |
| PSSJD_KI_01 | Cat. | 18-35 | F | Nurse technician |
| PSSJD_KI_02 | Cat. | 36-50 | F | Clinical psychologist |
| PSSJD_KI_03 | Cat. | 36-50 | F | Doctor |
| PSSJD_KI_04 | Cat. | 36-50 | M | Clinical psychologist |
| PSSJD_KI_05 | Cat. | 36-50 | F | Psychologist |
| PSSJD_KI_06 | Cat. | 18-35 | M | Psychologist |
| PSSJD_KI_07 | Cat. | 18-35 | F | Administrator |
| PSSJD_KI_08 | Cat. | 36-50 | F | Nurse supervisor |
| PSSJD_KI_09 | Cat. | 36-50 | F | Doctor |
| PSSJD_KI_10 | Cat. | 36-50 | F | Doctor |
| PSSJD_KI_11 | Cat. | 36-50 | M | Doctor |
| PSSJD_KI_12 | Cat. | 18-35 | M | Administrator |
| HULP_42 | Mad. | 18-35 | F | Nurse |
| HULP_43 | Mad. | > 50 | M | Nurse technician |
| HULP_44 | Mad. | 36-50 | M | Porter |
| HULP_45 | Mad. | 36-50 | F | Doctor |
| HULP_46 | Mad. | 36-50 | F | Nurse supervisor |
| HULP_47 | Mad. | 36-50 | F | Nurse technician |
| HULP_48 | Mad. | 18-35 | M | Psychiatrist |
| HULP_49 | Mad. | 18-35 | M | Mental health nurse |
| HULP_50 | Mad. | 36-50 | F | Psychiatrist |
| HULP_51 | Mad. | 36-50 | F | Nurse |

*Note.* *N* = 22 (*n* = 12, Catalonia; *n* = 10, Madrid). Cat. = Barcelona, Mad. = Madrid.
